# Genetic Mapping of Tolerance to Bacterial Stem Blight Caused by *Pseudomonas syringae* pv. *syringae* in Alfalfa (*Medicago sativa* L.)

**DOI:** 10.3390/plants13010110

**Published:** 2023-12-29

**Authors:** Yeidymar Sierra Moya, Cesar Medina, Bianca Herrera, Fabian Chamba, Long-Xi Yu, Zhanyou Xu, Deborah A. Samac

**Affiliations:** 1 Department of Plant Pathology, University of Minnesota, St. Paul, MN 55108, USA; sierr035@umn.edu (Y.S.M.); herre329@umn.edu (B.H.); 2 Department of Agronomy and Plant Genetics, University of Minnesota, St. Paul, MN 55108, USA; medin297@umn.edu; 3 Granja Barrera Inc., Stratford, ON N5A 2Y7, Canada; chamb376@umn.edu; 4 USDA-ARS-Plant Germplasm Introduction and Testing Research Unit, Prosser, WA 99350, USA; longxi.yu@usda.gov; 5 USDA-ARS-Plant Science Research Unit, St. Paul, MN 55108, USA; zhanyou.xu@usda.gov

**Keywords:** alfalfa, bacterial plant pathogen, bacterial stem blight, biparental F1 mapping population, plant disease, *Pseudomonas syringae*, QTL mapping, genotyping-by-sequencing

## Abstract

The bacterial stem blight of alfalfa (*Medicago sativa* L.), first reported in the United States in 1904, has emerged recently as a serious disease problem in the western states. The causal agent, *Pseudomonas syringae* pv. *syringae*, promotes frost damage and disease that can reduce first harvest yields by 50%. Resistant cultivars and an understanding of host-pathogen interactions are lacking in this pathosystem. With the goal of identifying DNA markers associated with disease resistance, we developed biparental F_1_ mapping populations using plants from the cultivar ZG9830. Leaflets of plants in the mapping populations were inoculated with a bacterial suspension using a needleless syringe and scored for disease symptoms. Bacterial populations were measured by culture plating and using a quantitative PCR assay. Surprisingly, leaflets with few to no symptoms had bacterial loads similar to leaflets with severe disease symptoms, indicating that plants without symptoms were tolerant to the bacterium. Genotyping-by-sequencing identified 11 significant SNP markers associated with the tolerance phenotype. This is the first study to identify DNA markers associated with tolerance to *P. syringae*. These results provide insight into host responses and provide markers that can be used in alfalfa breeding programs to develop improved cultivars to manage the bacterial stem blight of alfalfa.

## 1. Introduction

Alfalfa (*Medicago sativa* L.) is a key forage crop and plays an important role in sustainable agricultural systems in the United States [1]. Recently, bacterial stem blight (BSB) has been associated with increased disease and frost damage in alfalfa in central and western states, negatively impacting alfalfa biomass yield and the nutritive value of the crop [2]. Two bacteria, *Pseudomonas syringae* pv. *syringae* and *P. viridiflava,* have been shown to cause BSB symptoms in alfalfa [3]. *P. syringae* pv. *syringae* causing BSB is common in the central and western United States (Oregon, California, Colorado, Utah, Wyoming, Ohio, and Minnesota), and it has also been isolated in Australia, Europe, and western Iran [4,5]. The factors underlying the re-emergence of BSB are unknown; however, genetic analysis of the pathogens revealed that both bacteria are diverse and widespread, making a recent introduction of a more virulent pathogen unlikely [6]. The disease cycle is initiated when the surfaces of alfalfa plants become colonized by bacteria present in rain or from infected plant material [7]. Once in contact with the plant foliage, the bacteria multiply as epiphytes. Exposure to −4 °C is sufficient to cause frost damage that may be catalyzed by the ice nucleation activity of the bacterium, resulting in injury to the epidermis. The pathogen gains entry through the wounds, and disease symptoms appear 7 to 10 days after frost. Infected stems show water-soaked, yellowish to olive green lesions, commonly where the leaf is attached, and lesions extend to one side of the stem. Over time, stem lesions turn amber to black in color. Stems appear wilted and deformed, turn brown, and die. Lesions on leaves are first water-soaked, then chlorotic, often with interveinal chlorosis. As the disease progresses, leaves become necrotic and fall from the stem. The disease is limited to early spring and damage from BSB can lead to up to 50% of the total yield loss from the first harvest [7]. Currently, there are no resistant cultivars nor control measures for the disease.

*Pseudomonas syringae* is a model bacterium for understanding plant-pathogen interactions. Much of the current understanding of effector-triggered immunity (ETI) in plants was developed by studying the interactions of *Arabidopsis thaliana* with *P. syringae* [8,9,10]. Recognition of a pathogen effector protein by a host resistance gene-encoded protein activates a signaling cascade that leads to disease resistance, usually mediated by the rapid death of cells in the affected region to restrict the spread of the pathogen. Genetic studies have found resistance in crop plants against *Pseudomonas* species to be complex, regulated by several genes depending on the crop [11,12,13,14,15,16]. While there have been a variety of studies in legumes focused on disease resistance-related genes [16,17] and their effector signaling pathways [16,18,19,20], there have been few studies of specific genes that confer disease resistance in alfalfa.

In contrast to immune responses, plants can also exhibit tolerance to pathogens. Here, tolerance is defined as a response that reduces the negative effects of a high pathogen load on plant fitness. Tolerance has primarily been studied with fungal and oomycete pathogens [21], but has been identified in *A. thaliana* inoculated with the bacterial pathogens *P. syringae* pv. *tomato* [22,23], *P. viridiflava* [24,25], and *Xanthomonas campestris* [26,27]. Tolerance is considered to be a more stable trait than ETI when developing resistant cultivars but has not been widely used, mostly due to concerns that tolerant plants could increase inoculum potential [21]. Tolerance to *X. campestris* was found to be controlled by a single gene, *RXC1* [27], but no previous studies have been conducted to map tolerance to *Pseudomonas* species in any host plant.

Detecting and quantifying pathogen populations in infected material is crucial when monitoring disease severity and host responses [28]. While visual scoring has been widely used to evaluate disease resistance, symptoms do not provide information on the extent of pathogen colonization [28,29,30]. Culture-based methods are a sensitive technique for measuring bacterial populations but can be time-consuming and laborious [31,32]. To overcome this, molecular detection through quantitative PCR (qPCR) assays has been shown to be an excellent alternative by providing fast and high-throughput detection and quantification of microorganisms [33]. Several different primer sets have been developed for detecting and quantifying *P. syringae* [32,34,35]. The most sensitive qPCR assay was developed with primers QRT-ps16sV1-F (5′-ACGGGTACTTGTACCTGGTG-3′) and QRT-ps16sV2-R (5′-CGTTTCCGAGCGTTATCCC-3′) that amplify 87 bp of the 16S ribosomal DNA that is specific to *P. syringae* variable regions 1 and 2 [35]. The assay has a linear positive correlation of CFU/mg leaf tissue to 16S rRNA measurements, indicating that bacterial concentration can be calculated based on the quantity of 16S rRNA in a sample.

Previously, alfalfa cultivars varying in fall dormancy and winter survival were tested for susceptibility to *P. syringae* pv. *syringae* strain ALF3. The cultivars Maverick and ZG9830 were found to have up to 60% of plants with resistance to BSB, as determined by symptoms and bacterial populations in inoculated stem internodes [2]. Furthermore, transcriptome profiling of stem tissues from resistant and susceptible plants of Maverick and ZG9830 after inoculation showed that *P. syringae* pv. *syringae* activates the expression of defense response pathways and resistance genes [4]. Alfalfa is an obligate outcrossing highly heterogeneous autotetraploid (2n = 4x = 32), which makes genotyping a challenge. The use of full-sib F_1_ populations segregating for Verticillium wilt resistance combined with genotyping-by-sequencing has proven to be a successful approach to identifying disease resistance QTLs and candidate resistance genes in alfalfa [36]. With the goal of advancing our understanding of the genes involved in response to *P. syringae* pv. *syringae*, an individual plant showing disease symptoms (susceptible), and a plant with no disease symptoms after inoculation (resistant) were selected from ZG9830 and intercrossed to develop a biparental F_1_ mapping population. We used these populations to evaluate disease symptoms and bacterial load in leaves inoculated with *P. syringae* pv. *syringae* strain 1021. Surprisingly, inoculated leaves without symptoms or with slight disease symptoms had bacterial loads similar to those from plants with severe disease symptoms, which indicated that these plants exhibit tolerance to BSB. The populations were genotyped using genotyping-by-sequencing (GBS) to identify DNA markers and candidate genes associated with response to *P. syringae* pv. *syringae* strain 1021.

## 2. Results

### 2.1. Disease Phenotypes of F_1_ Populations

**Figure 1:**
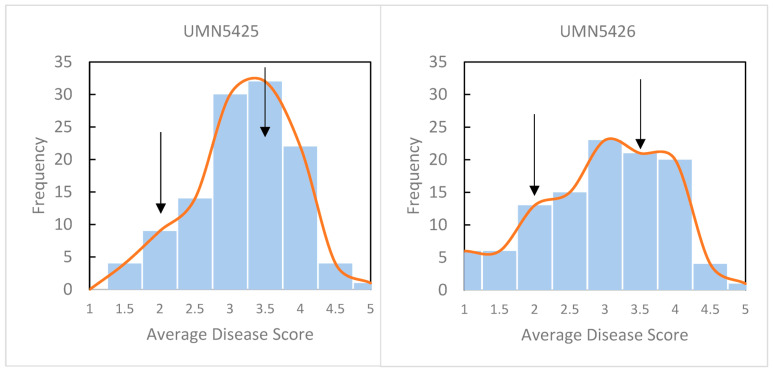
Histogram of disease scores from F_1_ and parental plants. Disease was scored on a 1 to 5 scale at 10 days post-inoculation after infiltration of leaflets with *P. syringae* pv. *syringae* strain 1021. 1 = no visible symptoms of infection; 2 = minimal chlorosis in the infiltrated area; 3 = necrosis with chlorosis in the infiltrated area; 4 = necrosis and chlorosis of entire leaflets, dehiscence of one or more leaflet; 5 = all leaflets necrotic and dried, dehiscence of two or more leaflets. Arrows indicate the average disease score of the resistant parent (2.0) and susceptible parent (3.5). A total of 116 plants were scored in UMN5425 and 109 plants in UMN5426.

Replicated disease phenotypes were obtained from 225 F_1_ plants at 10 days post-inoculation (dpi) with *P. syringae* pv. *syringae* strain 1021. A total of 116 plants were tested from UMN5425 developed from a cross with the susceptible plant as the female parent and 109 plants from UMN5426 with the resistant plant as the female parent. Disease symptoms were scored on a 1 to 5 scale where 1 = no symptoms and 5 = all leaflets necrotic and dried with dehiscence of two or more leaflets (see Materials and Methods for the complete disease scale). The scores from nine leaflets were averaged, and plants were categorized as resistant (mean score of 1 to 2), semi-resistant (>2 to 3), and susceptible (>3 to 5) (Figure 1). In total, 61 susceptible, 127 semi-resistant, and 37 resistant plants were identified. Estimates of means, range, standard deviation, coefficient of variation (CV), and broad sense heritability (H^2^) were calculated by population and for all F_1_ plants (Supplementary Table S1) [37]. Mean and CV values were not different among populations or for all F_1_ plants. Disease score means were 3.07 for UMN5425 and 2.79 for UMN5426, whereas CVs were 26% and 35% for UMN5425 and UMN5426, respectively. The value of heritability was high (80%), suggesting that the disease score is less affected by environmental influences.

**Figure 2:**
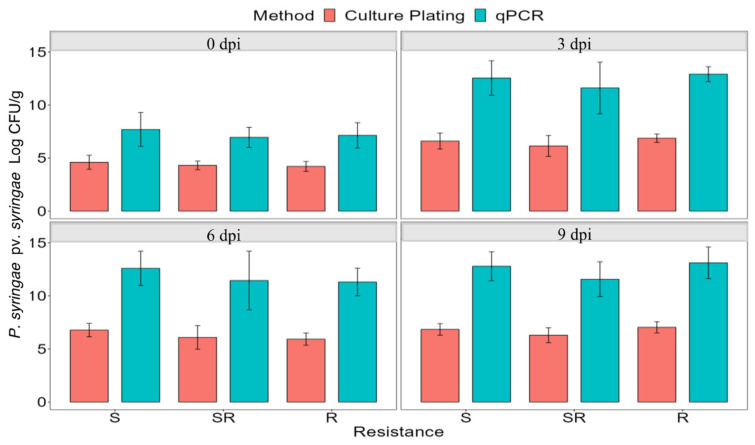
Comparison of average bacterial populations of *P. syringae* pv. *syringae* strain 1021 using qPCR and culture plating in resistant, semi-resistant, and susceptible plants immediately after inoculation (0 dpi), and 3, 6, and 9 dpi. The bars represent the average of nine leaflets and the standard deviation of the mean. dpi = days post-inoculation.

A total of 72 plants, scored as susceptible (45), semi-resistant (18), or resistant (9) from the F_1_ populations were clonally propagated, inoculated with strain 1021, and bacterial CFUs determined by culture plating and qPCR at 0, 3, 6, and 9 dpi (Figure 2). Quantification through culture plating had a range of approximately 10^6^ to 10^9^ CFU/g leaflets. In contrast, higher quantities of bacteria were measured in the qPCR assay, most likely because the assay measures both viable and non-viable bacteria. Bacterial populations increased significantly in all categories of plants from 0 to 3 dpi but did not increase significantly at 6 and 9 dpi. At each time point, there was no significant difference in bacterial load among susceptible, semi-resistant, or resistant plants, as determined by a one-way ANOVA (*p* < 0.05) (Figure 2). Based on these results, symptom severity in alfalfa leaves was not associated with the level of bacterial load in the infected tissue.

### 2.2. Genotyping Coverage and Population Structure

**Figure 3:**
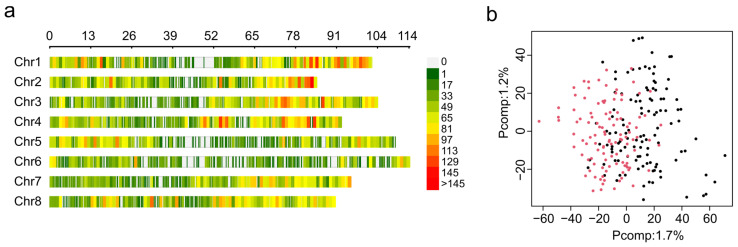
Single nucleotide polymorphism sites (SNPs) identified in the F_1_ mapping population. (**a**) Distribution of SNP markers across eight alfalfa chromosomes using a 0.1 Mb window size. The colored lines represent the marker density, as shown on the right color legend. (**b**) Principal component analysis (PCA) of SNPs. The plot was colored by subpopulations, with UMN5425 in black and UMN5426 in red.

The DNA from 242 plants was used for GBS, but during the process of acquiring phenotypic data, only 210 plants survived. Thus, the analysis was conducted only for plants with data from both genotyping and phenotyping. In total, 374,619,320 raw reads were obtained via GBS, and the overall alignment rate was 82% to the alfalfa reference genome of cultivar Zhongmu No. 1 [38]. After filtering, 28,346 high-quality SNP markers were obtained and plotted according to their position in the alfalfa genome using a 0.1 Mb window size (Figure 3a). The distribution of markers by chromosome was as follows: Chr. 1 = 3978 markers, Chr. 2 = 3621 markers, Chr. 3 = 3753 markers, Chr. 4 = 3922 markers, Chr. 5 = 3213 markers, Chr. 6 = 2558 markers, Chr. 7 = 3482 markers, and Chr. 8 = 3819 markers. Although the number of markers was similar among chromosomes, their distribution across the chromosomes was not uniform and resulted in gaps in the coverage of some chromosomes, such as 1, 5, and 6 (Figure 3a).

GWAS analysis is affected by the presence of population structure. Therefore, 28,346 high-quality SNP markers were used for genetic structure using principal component analysis (PCA). The PCA scree plot indicates that the first ten PCs explain 11.4% of the total genetic variance, and the first and second principal components were able to explain 1.7% and 1.2% of genetic variance, respectively (Supplementary Figure S1). PCA was not able to detect population stratification between F_1_ mapping populations UMN5425 and UMN5426 (Figure 3b). This result demonstrates that using the ZG21 plant as the female or male parent did not affect the genetic variance. For GWAS analysis, we included population structure information as fixed factors using the three first principal components to control false positive associations.

### 2.3. Genome-Wide Association Studies

Genome-Wide Association Studies (GWAS) were performed by combining phenotypic data and genotypic data. Disease scores were collected from 210 individuals. The additive GWASpoly analysis identified 11 non-redundant markers that are present within gene regions linked with tolerance (Table 1). Potential candidate genes linked to marker loci related to BSB tolerance were identified by conducting a BLAST search against the UniProt database.

**Figure 4:**
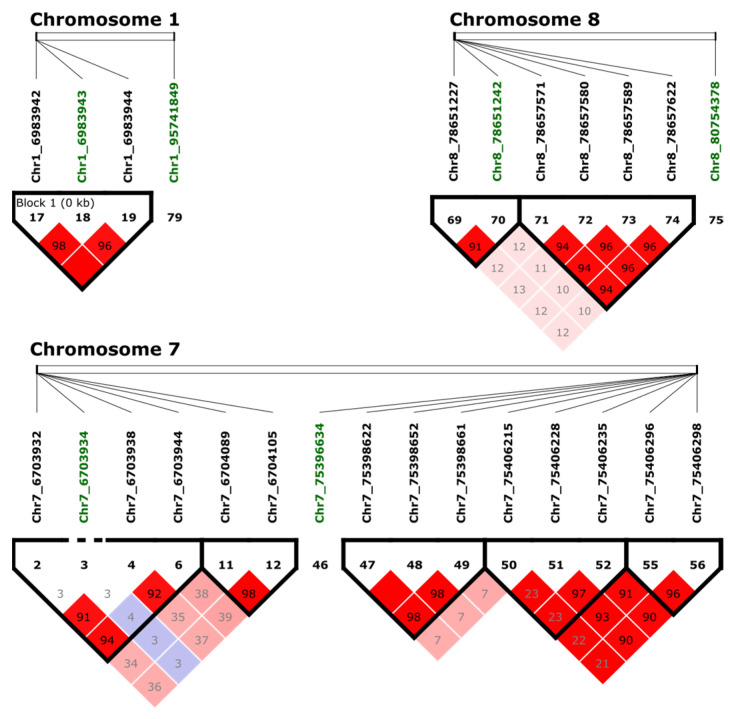
Haploview analysis and pairwise measures of LD between the SNP of interest (in green) and adjacent markers. The black line at the top of the figure represents a region of the genome with four lines that represent the relative physical position of four SNPs. Numbers within the red squares represents the scores (D′) of the pairwise LD between SNPs and a red squared with no number indicates complete LD (100). Bright red indicates *D*′ = 1, *LOD* ≥ 2; blue coloring indicates *D*′ = 1, *LOD* < 2; white coloring indicates *D*′ < 1, *LOD* < 2; shades of pink/red coloring indicate *D*′ < 1, *LOD* ≥ 2.

From the 11 significant markers, one marker based on the BLAST search and annotation, Chr1_6983943, was located within a disease-resistance protein with an NB-ARC domain. Using the genome sequence and transcriptome information, putative exons were identified for this gene (Supplementary Figure S2a). Two adjacent SNPs, Chr1_6983942 and Chr1_6983944, were identified and included in the haplotype analysis (Figure 4). SNPs Chr1_6983942 and Chr1_6983943 were the second and first nucleotides in codon 481. The reference haplotype (GGT) codes for glycine (Gly), but changes in one or both SNPs will change the amino acid to cysteine (Cys), alanine (Ala), or serine (Ser) (Supplementary Figure S2b). SNP Chr1_6983944 had no effect on the amino acid sequence.

### 2.4. Examining Linkage Disequilibrium (LD)

LD analysis among markers associated with tolerance to BSB was performed using adjacent SNPs in a 10 kb window by Haploview v4.2 [37]. Three blocks were identified with significant SNPs (Figure 4). One block in Chr1 with three adjacent SNPs (Chr1_6983942, Chr1_6983943, and Chr1_6983944) was identified. The extent of LD in a genome can be statistically quantified, allowing a better understanding of genomic variations [38]. The linkage score of Chr1_6983942 and Chr1_6983943 was 98%, and the linkage score of Chr1_6983943 and Chr1_6983944 was 96%. Interestingly, based on the Pearson’s correlation, the pairwise comparison between the markers Chr1_6983942 and Chr1_6983943 showed a strong LD with a correlation of 81%, while Chr1_6983944 compared with Chr1_6983942 correlated 51%, and Chr1_6983943 had a correlation of 53%. Based on these findings, there are three SNPs within the same region of the chromosome, but only Chr1_6983942 and Chr1_6983943 had a significant LD. In Chr7, the markers Chr7_6703932, Chr7_6703938, and Chr7_6703944 are in LD, but they were not in LD with the significant marker Chr7_6703934. One block was identified in Chr8 with two markers (Chr8_78651227 and Chr8_78651242). Additional significant associated markers were not in LD with adjacent markers.

To understand how allelic dosage affects the BSB disease score, linear regression was performed between the phenotypic data and the allele dosage of the markers Chr1_6983943 and Chr5_19814514 (Supplementary Figure S3). There was a significant effect of the allele dosage on the BSB disease score for both markers (*p*-value > 0.01). For Chr1_6983943, the effect (R) of −0.24 implies that each change from reference to alternative allele in this locus will decrease the disease score by 0.24. For Chr5_19814514, the R of 0.23 implies increased susceptibility with each allele change. For the marker Chr1_6983943, there were no genotyped individuals with all reference SNPs (CCCC).

## 3. Discussion

Bacterial stem blight in alfalfa has both a stem lesion with vascular wilt disease phenotype and a leaf blight disease phenotype, depending on the site of infection. This study rated disease symptoms and measured populations of *P. syringae* pv. *syringae* after infiltration of the bacterium into leaves of plants in an F_1_ mapping population. This population was developed from plants found to be resistant or susceptible after stem inoculation and measurement of bacterial populations in inoculated stems. In the F_1_ population, alfalfa leaves scored as “resistant” with few to no symptoms at 9 dpi and had similar amounts of bacteria as those with severe symptoms, indicating that these plants show tolerance to the foliar phase of BSB.

In the majority of *P. syringae* pathosystems, there is a strong positive correlation between symptoms and CFUs [22,23]; that is, plants with more severe disease symptoms have higher CFUs than plants with mild or no visible symptoms. Resistant plants are generally capable of reducing or eliminating pathogen infection, growth, and reproduction. The phenomenon of tolerance, the capacity of a plant to survive, reproduce, and adapt to disease has received less attention. One study analyzed the difference in pathogen growth, disease symptoms, and host fitness in *A. thaliana* and *P. syringae* pv. *tomato* strain DC3000 to determine the importance of resistance and tolerance and to understand the effects of covariance [22]. The results showed a quantitative variation in bacterial population size, severity of symptoms, and the effects of infection on plant fitness. There was a strong positive correlation between bacterial population size and disease symptoms, but no correlation between bacterial growth or symptoms and relative fitness after infection as measured by seed production [22]. In other words, their results suggest the presence of genetic polymorphisms for tolerance and the coexistence of tolerance and resistance traits in the same plant genotype [21]. Other studies [24,25] found similar results with the interaction of *A. thaliana* and *P. viridiflava*. This might imply that, like the interaction of *A. thaliana* and *P. syringae*, other pathosystems might show tolerance against a pathogen rather than resistance, and that we cannot predict host fitness based on the severity of symptoms and bacterial load [22].

Significant variation in tolerance opens new opportunities to assess the genetic basis for tolerance and thus facilitate disease control strategies. In the *P. syringae* and alfalfa pathosystem, plant fitness was not measured directly. However, the retention of leaves in tolerant plants that had high bacterial loads would likely result in higher biomass yields and possibly higher seed yields. Developing durable disease resistance is a major goal in plant breeding. Introducing resistance genes into susceptible cultivars often breaks down rapidly since pathogens can adapt to and overcome them. Tolerance may be an effective means to reduce damage from disease, although, for polycyclic diseases, tolerant plants could increase the inoculum in the environment.

*P. syringae* has an extensive arsenal for causing plant disease [49]. Among these are phytotoxins causing chlorosis and necrosis, and some inhibit host defenses [50,51]. One of these is coronatine (COR), a nonhost-specific virulence factor with structures and functions like jasmonate and jasmonic acid-isoleucine [50,52]. This phytotoxin can mimic and disrupt important plant hormones in cellular defense response pathways, suppress the salicylic acid (SA) pathway and closure of stomata, and allow the entrance of more pathogenic bacteria, promoting lesion expansion and bacterial growth. Even though multiple studies have used COR to understand phytotoxins, there is still a gap in knowledge on the specific mechanisms used by COR to promote bacterial virulence and disease symptomatology. In the alfalfa genotypes that do not show disease symptoms in response to infection by *P. syringae*, a possible tolerance mechanism could be insensitivity to one or more phytotoxins. Interestingly, the isolates of *P. syringae* pv. *syringae* causing BSB in alfalfa have genes to produce coronatine (COR), which is unusual in this pathovar [53]. Thus, the sensitivity of tolerant alfalfa plants to COR and other phytotoxins produced by *P. syringae* pv. *syringae* strain 1021 warrants further investigation.

In *P. syringae* pv. *syringae,* N-acyl homoserine lactone (AHL) is the quorum sensing (QS) signal molecule, and high concentrations of AHL result in the expression of secondary metabolites and virulence factors that facilitate effective colonization in the host and disease [54]. In other pathosystems, plants have been able to derive molecules with a quorum sensing inhibitor (QSI) function used to defeat QS pathogens [55]. Some QSI can stop the synthesis of AHL, degrade the signal molecule, or target its receptor [55], and plants can produce several compounds that can target different bacterial strains [56]. Alfalfa has been found to have AHL-degrading abilities [55], although its expression in the plants used in these experiments is unknown. Tolerant alfalfa plants did not exhibit water-soaking, a QS-dependent trait after inoculation and colonization by *P. syringae* pv. *syringae* strain 1021, suggesting a possible mechanism underpinning the tolerance observed.

Few studies have mapped QTLs for tolerance in plant-pathogen interactions. In this study, we used quantitative approaches for gene function prediction, linking phenotypic data and genotypic data, suggesting that statistically significant variation at the gene locus of an SNP is responsible for or associated with the tolerance response. As discussed by Miles and Wayne (2008) [57], the combination of QTL and GWAS analysis can provide a powerful approach and achieve a higher magnitude of resolution to elucidate genes or nucleotides that contribute to the phenotype of interest. We identified 11 significant SNP markers associated with tolerance to BSB. All of these markers were in coding regions with different reported functions, and some of the most relevant are discussed below. Marker Chr4_77596753 was located in a locus annotated as *GAD* involved in the accumulation of gamma-aminobutyric acid (GABA) by the alpha-decarboxylation of glutamate to gamma-aminobutyrate [58]. GABA accumulation increases tolerance to abiotic stress, inhibiting reactive oxygen species (ROS) generation, decreasing ionic accumulation, or regulating stomatal opening. GAD activity is dependent on cytosolic calcium levels [59]. GABA is also involved in the biotic stress response, limiting cell death caused by excessive ROS. GABA levels increase in the interaction of *Phaseolus vulgaris* with *P. syringae* pv. *phaseolicola* [60] or *A. thaliana* with *P. syringae* pv. *tomato* (*Pto*) DC3000 [61]. Deng et al. 2020 [42] found that *GAD1, GAD2,* and *GAD4* play a positive role in both PTI and ETI through the activation of the MPK3/MPK6 signaling cascade in *A. thaliana* with *Pto* DC3000 [42].

Two markers were located at loci involved in plant defense: Chr1_6983943 was located in an NB-ARC domain disease resistance gene, and Chr5_19814514 was located in *SNL6* [43,62]. NB-ARC proteins are key components of the plant immune system. The NB-ARC proteins contain a central nucleotide-binding domain and two ARC domains with a conserved HMD motif. The NB domain functions as an NTP-hydrolyzing switch, regulating signal transduction through conformational changes [63]. The marker Chr1_6983943 produces a change in Gly481, 20 amino acids downstream of the HMD motif (461–463). Previous reports of NB-ARC as a disease resistance protein include the expression of *VpCN* from *Vitis pseudoreticulata* in *A. thaliana* to enhance the resistance to *Golovinomyces cichoracearum* and *Pto* DC3000 [64] or the mapping of a NB-ARC gene in the interaction between *Medicago truncaltula* and *Aphanomyces euteiches* [38]. To date, there are no reports of resistance genes in the interaction of alfalfa and *P. syringae* pv. *syringae*, but further analysis can validate the role of this NB-ARC gene in resistance/tolerance to BSB.

*SNL6* encodes a cinnamoyl-CoA reductase-like (CCR) protein with a role in tissue lignification. The *snl6* mutant lines in rice have a lower lignin content with increased sugar extractability [43]. In alfalfa, CCR down-regulated lines had significantly enhanced saccharification efficiency [65]. However, *SNL6* is a suppressor of NH1-mediated lesion formation. The *snl6* mutant lines in rice fail to develop the NH1-mediated lesions, PR gene activation, and resistance to *Xanthomonas oryzae* pv. *oryzae* [43]. Interestingly, three markers, Chr5_34806446, Chr7_6703934, and Chr8_78651242 were located at loci annotated as *UGD*, *PE*, and *GT-BC10,* with roles in cell wall biosynthesis and cell wall stability [44,45,46,47]. Cell walls have an active role in plant defense as a physical barrier or as signaling molecules triggering plant immune responses and there can be additional modifications involved in the PTI. Future experiments can employ molecular techniques to narrow down the QTLs to more specific candidate genes. Additionally, the linkage and association studies might allow the development of molecular markers in breeding programs for the transfer of tolerance to BSB to novel alfalfa varieties.

## 4. Materials and Methods

### 4.1. Bacterial Culture Conditions

The *P. syringae* pv. *syringae* strain 1021 was isolated from an alfalfa plant with bacterial stem blight symptoms in Scott Valley, CA, USA, in 2017. Preliminary experiments tested six strains isolated from five locations over a period of five years, with individual plants selected from ZG9830 [66]. There was no strong evidence for strain-host plant specificity. Plant responses to strain 1021 were reproducible and the strain was highly pathogenic on susceptible alfalfa plants. Bacteria were stored at −80 °C in 20% glycerol. Prior to inoculation, the culture was revived on King’s B medium, then a single colony was used to inoculate 5 mL Nutrient Broth Yeast extract medium and cultured for 24 h at 25 °C in an orbital shaking incubator at 250 rpm. The bacterial suspension was pelleted by centrifugation for 5 min at 3500 rpm and resuspended in sterile 10 mM KPO_4_ buffer, with a pH of 7 to obtain bacterial suspensions at a final optical cell density (OD_600_) of 0.05, approximately 5 × 10^7^ CFU/mL.

### 4.2. Development of Mapping Populations and Plant Growth Conditions

Biparental crosses were made by hand pollination between individual resistant (ZG21) and susceptible plants (ZG25) selected from ZG9830 [4] and F_1_ seeds were collected from the female parents. Seeds of the populations UMN5425 (ZG25 as the female parent and ZG21 as the male parent) and UMN5426 (ZG21 as the female parent and ZG25 as the male parent) were sandpaper scarified, planted in pasteurized greenhouse soil in SC1OU cone-tainers (Stuewe and Sons, Tangent, OR, USA), and maintained in a greenhouse with a 16 h photoperiod. Vegetative cuttings of each F_1_ plant were made by rooting stem segments in medium grade vermiculite. Cuttings were transplanted into a mixture of SunGro Germination Mix (SunGro Horticulture, Agawam, MA, USA) and pasteurized greenhouse soil and maintained in a growth chamber with a 16 h/8 h (light/dark) photoperiod at 25 °C.

### 4.3. Leaf Inoculation and Disease Scoring

Fully expanded leaves at the second or third node from the top of the stem from growth chamber-grown plants were tagged with cotton yarn on the petiole for inoculation. Each of the three leaflets of a leaf was infiltrated from the abaxial side with approximately 0.1 mL of a bacterial suspension at OD_600_ = 0.05 using a needle-less syringe. The negative control was infiltrated using 10 mM KPO_4_ buffer, pH 7. After inoculation, the plants were returned to the growth chamber. For each F_1_ plant, three leaves (nine leaflets) were inoculated and used for pathogen quantification.

**Figure 5:**
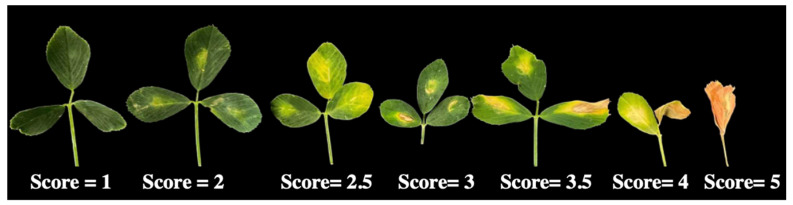
The scoring system used to evaluate bacterial stem blight disease severity in alfalfa leaves 10 days after leaf infiltration with *P. syringae* pv. *syringae* strain 1021.

Symptoms were scored visually at 10 days after inoculation on a 1 to 5 scale (Figure 5); 1 = no visible symptoms of infection; 2 = minimal chlorosis in the infiltrated area; 2.5 = chlorosis extending beyond the infiltrated area; 3 = necrosis with chlorosis in the infiltrated area; 3.5 = necrosis and chlorosis extending beyond the infiltrated area; 4 = necrosis and chlorosis of entire leaflets, dehiscence of one or more leaflet; 5 = all leaflets necrotic and dried, dehiscence of two or more leaflets. Disease scores from nine leaflets were averaged and plants were categorized as resistant (1 to 2), semi-resistant (>2 to 3), or susceptible (>3 to 5).

### 4.4. Pathogen Quantification

Inoculated leaflets were removed from plants and weighed individually. Each leaflet was placed in a FastPrep^®^ 2 mL Lysing Matrix tube (MP Biomedicals, Solon, OH, USA) containing ceramic spheres (6.35 mm) and 0.7 mL of 10 mM KPO_4_ buffer, pH 7. Samples were homogenized using an MP Biomedicals™ FastPrep™ high-speed benchtop homogenizer. Homogenized material was transferred into a microfuge tube, and 10 mM KPO_4_ buffer was added to a final volume of 1 mL. Samples were diluted by ten-fold serial dilutions. To determine CFUs, 10 μL of the sample dilutions were plated in triplicate on KB agar medium and incubated at room temperature. After 28 h, plates were placed under UV light to confirm the presence of *P. syringae*, and CFUs determined.

Each qPCR assay (20 µL) consisted of 5 µL bacterial cells, 1 µL primer forward (10 µM), 1 µL primer reverse (10 µM), 3 µL nuclease-free water, and 10 µL SsoAdvanced Universal SYBR Green Supermix (BIORAD Laboratories, Inc., Hercules, CA, USA). The primers utilized were QRT-ps16sV1-F (5′-ACG GGTACTTGTACCTGGTG-3′) and QRT-ps16sV2-R (5′-CGTTTCCGAGCGTTATCCC-3′) that amplify an 87 bp portion of the 16S ribosomal subunit (*Ps16S* gene; PSPTO_r01) [35]. Assays were conducted using an Applied Biosystems 7500 Fast Real-Time PCR System (ThermoFisher Scientific, Waltham, MA, USA) for 40 cycles with initial polymerase activation for 2 min at 98 °C followed by denaturation at 98 °C for 15 s, then annealing/extension at 60 °C for 60 s. The melting curve analysis was performed using the instrument’s default setting after the final cycle. A standard curve was generated using dilutions of *P. syringae* pv. *syringae* strain 1021 cells by plotting the cycle threshold value (Ct) for each sample of the standard series versus the logarithm of bacterial concentration (CFU/mL) as determined by culture plating.

### 4.5. DNA Extraction and Sequencing

Young leaves were harvested from 242 plants and DNA was extracted using the Qiagen DNeasy 96 Plant Kit (Qiagen, Valencia, CA, USA) following the manufacturer’s instructions. A NanoDrop 2000 spectrophotometer (NanoDrop Technologies, Inc. Wilmington, DE, USA) was used for measuring DNA concentration and quality. The extracted DNA was submitted to the University of Minnesota Genomics Center for processing and sequencing using the GBS protocol according to Elshire et al. [67]. Briefly, genomic DNA (100 ng) was digested with 10 units of *Ape*KI (NEB) and incubated at 75 °C for 2 h. Phased adaptors with a three-base overhang on the 5′ ends of the bottom strand were ligated with digested DNA and 200 units of T4 ligase (NEB) at 22 °C for 1 h and heat-inactivated. The ligated samples were purified, and bar codes were added by 18 cycles with 2X NEB Taq Master Mix. Lastly, pooled libraries were size-selected for the 300 to 744 bp library region (156 to 600 DNA inserts). The final pool was then diluted to 1 nM and sequenced on the Illumina NovaSeq 6000 using single-end 1 × 100 reads on a single lane of an SP variant flowcell.

### 4.6. GBS and Variant Calling

The raw sequencing data (fastq files) were cleaned using fastp software v0.23.4 [68] before being aligned to the alfalfa genome [38] using the Next Generation Sequencing Experience Platform (NGSEP) software v4.2.0 [69] and the function ReadsAligner to generate Binary Alignment Map (BAM) files. BAM files were sorted using Picard tools software v3.1.1 with default parameters [70]. Variants were called with the function MultisampleVariantDetector of NGSEP software v4.2.0 controlling the PCR duplicates of GBS samples increasing the maximum number of alignments allowed to start at the same reference site (maxAlnsPerStartPos) to 100 to retain high sensitivity generating a variant call format (VCF) file. The VCF file was filtered using the function VCFFilter of NGSEP software v4.2.0 as follows: (i) maximum value allowed for a base quality score: 30; (ii) minimum allele frequency of 0.05; (iii) maintained positions at least 70% of the samples are genotyped; (iv) minimum genotyping quality 40; (v) ploidy = 4; (vi) imputation using hidden Markov model implemented in NGSEP v4.0.0. After filtering, 28,346 high quality biallelic SNP markers were obtained and transformed into GWASpoly format [67] using the function VCFConverter of NGSEP software v4.2.0 [69].

### 4.7. Association Mapping and Annotation

The genome-wide association studies were performed using the R package GWASpoly using a Q+K mixed linear model that incorporates three principal components as population structure (Q) and a kinship matrix (K) as follows [71]:

y=Xβ+ZSτ+ZQv+Zu+ε

where y corresponds to the observed phenotypes; X is an incidence matrix of fixed effects; β is a vector of fixed effects; Z is a matrix of incidence mapping genotypes to phenotypes; S is a structure incidence matrix; τ is a SNPs effects vector; Q is an incidence matrix for population size; v is the subpopulations vector effects; u is a polygenic effects vector; and ε is a residuals vector [71]. Finally, markers associated with BSB tolerance were identified using a threshold False Discovery Rate (FDR) < 0.05 [72], and the candidate loci were annotated using the *M. sativa* cv. Zhongmu1 genome [18] and the alfalfa pan-transcriptome [73] were corroborated by BLAST. Coding regions located in the candidate loci were annotated using Uniprot ID and the protein function was retrieved using a bibliographical search.

## Figures and Tables

**Table 1:** Significant markers associated with tolerance to bacterial stem blight. SNP shows the reference and alternative nucleotides. R^2^ is the percentage of explained variance for each marker; Effect is the effect of the nucleotide substitution on BSB disease score.

Marker	SNP	−log *p*-Value	R^2^	Effect	Uniprot ID	Gene	Annotation	Role	Reference
Chr1_6983943	C/A	3.47	0.056	−0.257	G7J119	−	NB-ARC domain disease resistance protein	Response to biotic stress	[39]
Chr1_95741849	G/A	3.4	0.051	0.251	UPI000B7B775A	*NAT4*	Nucleobase-ascorbate transporter 4	Response to abiotic stress	[40]
Chr2_16508382	T/C	3.73	0.028	0.219	W4ZQ03	−	Bifunctional inhibitor/plant lipid transfer protein/seed storage helical domain-containing protein	−	
Chr3_75660736	C/G	4.15	0.033	0.404	UPI0011DFD893	−	RING-type E3 ubiquitin transferase	Plant growth and response to abiotic stress	[41]
Chr4_77596753	T/A	3.36	0.037	−0.266	UPI001016EB81	*GAD*	Glutamate decarboxylase	Response to biotic stress	[42]
Chr5_19814514	G/A	3.45	0.031	0.214	A0A445LXJ0	*Snl6*	Cinnamoyl-CoA reductase-like SNL6	Response to biotic stress	[43]
Chr5_34806446	A/G	3.31	0.008	0.220	A0A061EAW6	*UGD*	UDP-glucose 6-dehydrogenase	Cell wall biosynthesis	[44]
Chr7_6703934	T/C	3.38	0.076	0.218	A0A059BED4	*PE*	Pectinesterase	Cell wall stability	[45]
Chr7_75396634	A/T	3.87	0.029	0.343	A0A445CS76	*PSEN*	Presenilin	Protein cleavage	[46]
Chr8_78651242	C/T	3.41	0.032	−0.195	A0A371G107	*GT-BC10*	Glycosyltransferase BC10	Biosynthesis of polysaccharides and glycoproteins	[47]
Chr8_80754378	T/G	3.32	0.032	−0.314	A0A445DK30	−	Protein kinase domain-containing protein	Plant signaling and response to abiotic stress	[48]

## Data Availability

The data presented in this study are available on request from the corresponding author.

## Supplementary Materials

The following supporting information can be downloaded at: https://www.mdpi.com/article/10.3390/plants13010110/s1, Figure S1: Principal component analysis (PCA) scree plot of the first ten principal components generated with 28,346 high-quality SNP markers, Figure S2: Diagram depicting the NB-ARC disease resistance gene in alfalfa using *M. sativa* cv. Zhongmu No. 1 as reference genome, Figure S3: Allele-specific analysis of the markers Chr1_6983943 and Chr5_19814514, Table S1: Phenotypic variation of disease score. Mean, range, standard deviation (SD), and coefficient of variation (CV) were calculated by population and for F_1_ plants.
