# Supplementary material for: Genetic Mapping of Tolerance to Bacterial Stem Blight Caused by Pseudomonas syringae pv. syringae in Alfalfa (Medicago sativa L.)
Source: Plants (Basel). 2023 Dec 29;13(1):110. doi: 10.3390/plants13010110 (PMC10780931; doi:10.3390/plants13010110)
Supplement: Supplementary file 1 [file plants-13-00110-s001.zip › plants-2726109-supplementary.pdf]

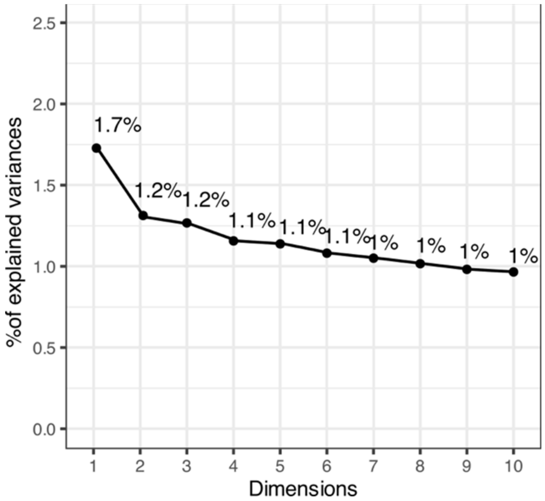

Supplementary Figure S1. Principal component analysis (PCA) scree plot of the first ten principal components generated with 28,346 high-quality SNP markers.

a

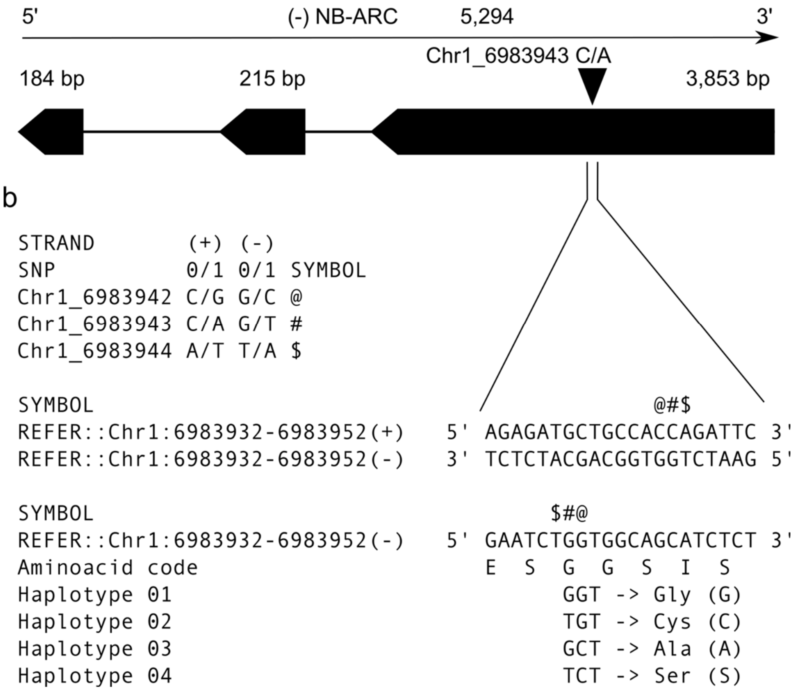

b

```
STRAND         (+) (-)
SNP            0/1 0/1 SYMBOL
Chr1_6983942 C/G G/C @
Chr1_6983943 C/A G/T #
Chr1_6983944 A/T T/A $

SYMBOL                                               @#$
REFER::Chr1:6983932-6983952(+)    5' AGAGATGCTGCCACCAGATTC 3'
REFER::Chr1:6983932-6983952(-)    3' TCTCTACGACGGTGGTCTAAG 5'

SYMBOL                                      $#@
REFER::Chr1:6983932-6983952(-)    5' GAATCTGGTGGCAGCATCTCT 3'
Aminoacid code                        E  S  G  G  S  I  S
Haplotype 01                                GGT -> Gly (G)
Haplotype 02                                TGT -> Cys (C)
Haplotype 03                                GCT -> Ala (A)
Haplotype 04                                TCT -> Ser (S)
```

Supplementary Figure S2. Diagram depicting the NB-ARC disease resistance gene in alfalfa using *M. sativa* cv. Zhongmu No. 1 as reference genome [38]. (**a**) The coding region encompasses 5,294 bp and contains three exons represented by black boxes. Numbers above the black boxes indicate the exon length in base pairs. The black arrow over the first exon indicates the approximate location of the SNP Chr1_6983943, indicating the reference and alternative bases in the alfalfa genome. (**b**) The NB-ARC disease resistance gene is coded on the negative strand changing the reference and alternative bases (0/1). Two additional SNPs were in the same locus: Chr1_6983942 and Chr1_6983944. Changes in SNPs Chr1_6983942 and Chr1_6983943 produce four haplotypes.

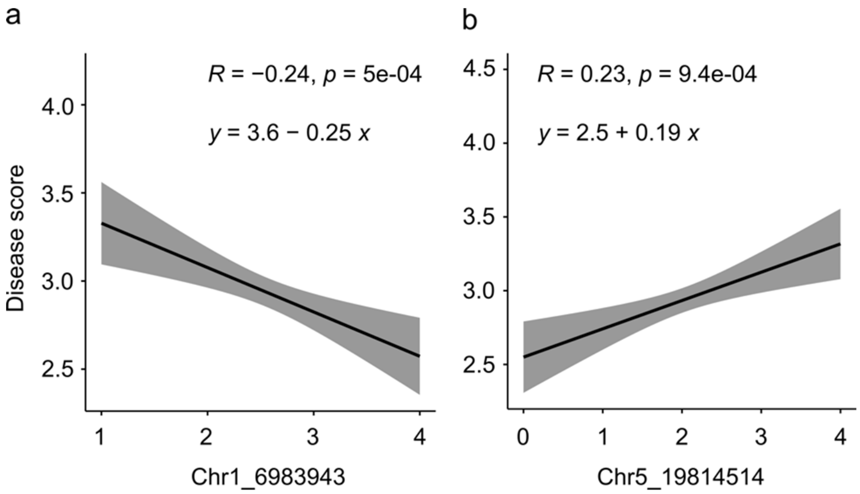

Supplementary Figure S3: Allele-specific analysis of the markers Chr1_6983943 and Chr5_19814514. Linear regression was used to quantify the relative impacts of additive genetic effects of allele dosage and disease score. The y-axis represents the mean of the disease score, and the x-axis represents the allele dosage. (**a**) Alleles of the marker Chr1_6983943 are 1 = CCCA, 2 = CCAA, 3 = CAAA, 4 = AAAA. (**b**) Alleles of the marker Chr5_19814514 are 0 = GGGG 1 = GGGA, 2 = GGAA, 3 = GAAA, 4 = AAAA.

Supplementary Table S1. Phenotypic variation of disease score. Mean, range, standard deviation (SD) and coefficient of variation (CV) were calculated by population and for F_1 plants. Broad sense heritability (H^2) was calculated for all samples using the Cullis method [37] assuming the genetic term as random effect.

Pop	Mean	Range	SD	CV	H^2
5425	2.83	1-5	0.96	0.34	–
5426	2.70	1-5	1.09	0.40	–
all	2.76	1-5	1.03	0.37	0.80
